# (2,2′-Bipyridine-κ^2^ *N*,*N*′)bis­(4-formyl­benzoato-κ*O* ^1^)copper(II)

**DOI:** 10.1107/S1600536812031066

**Published:** 2012-07-14

**Authors:** Jin-li Qi, Wei Xu

**Affiliations:** a Center of Applied Solid State Chemistry Research, Ningbo University, Ningbo, Zhejiang 315211, People’s Republic of China

## Abstract

The title mononuclear Cu^II^ complex, [Cu(C_8_H_5_O_3_)_2_(C_10_H_8_N_2_)], is comprised of a Cu^II^ cation, two 4-formyl­benzoate (*L* ^−^) ligands and a 2,2′-bipyridine (bipy) ligand. The Cu^II^ ion and bipy ligand lie on a crystallographic twofold rotation axis; the Cu^II^ ion is coordinated by two N atoms from one bipy ligand and two O atoms from two different carboxyl­ate groups of two *L* ^−^ ligands, exhibiting effectively a distorted square-planar geometry. The complex mol­ecules are inter­linked to generate two-dimensional supra­molecular layers in the *ab* plane, formed by C—H⋯O hydrogen bonds, where the O acceptor is the O atom from the carboxyl­ate group not involved in coordination to the Cu^II^ ion. The two-dimensional layers are stacked in a sequence *via* C—H⋯O hydrogen-bonding inter­actions where the formyl O atom acts as acceptor.

## Related literature

For general background on the use of transition metal complexes containing carboxyl­ate ligands and secondary building units, see: Sun *et al.* (2002 ▶); Liu *et al.* (2006 ▶); Xu *et al.* (2011 ▶). For related structures using the same metal, similar ligands and with a similar coordination environment, see: Li *et al.* (2007 ▶).
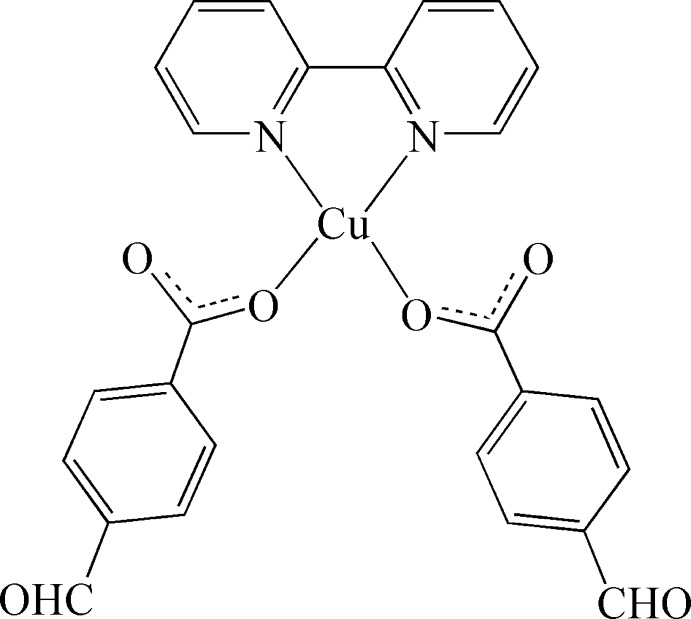


## Experimental

### 

#### Crystal data

[Cu(C_8_H_5_O_3_)_2_(C_10_H_8_N_2_)]
*M* *_r_* = 517.96
Monoclinic, 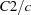

*a* = 11.923 (2) Å
*b* = 10.992 (2) Å
*c* = 18.275 (4) Å
β = 100.11 (3)°
*V* = 2357.9 (8) Å^3^
*Z* = 4
Mo *K*α radiation
μ = 0.97 mm^−1^
*T* = 295 K
0.23 × 0.17 × 0.08 mm

#### Data collection

Rigaku R-AXIS RAPID diffractometer
Absorption correction: empirical (using intensity measurements) (*ABSCOR*; Higashi, 1995 ▶) *T*_min_ = 0.820, *T*_max_ = 0.925
11333 measured reflections
2700 independent reflections
1672 reflections with *I* > 2σ(*I*)
*R*_int_ = 0.067

#### Refinement

*R*[*F* ^2^ > 2σ(*F* ^2^)] = 0.058
*wR*(*F* ^2^) = 0.146
*S* = 1.23
2698 reflections
159 parameters
H-atom parameters constrained
Δρ_max_ = 1.16 e Å^−3^
Δρ_min_ = −0.51 e Å^−3^

### 

Data collection: *RAPID-AUTO* (Rigaku, 1998 ▶); cell refinement: *RAPID-AUTO*; data reduction: *CrystalStructure* (Rigaku/MSC, 2004 ▶); program(s) used to solve structure: *SHELXS97* (Sheldrick, 2008 ▶); program(s) used to refine structure: *SHELXL97* (Sheldrick, 2008 ▶); molecular graphics: *ORTEPII* (Johnson, 1976 ▶) and *DIAMOND* (Brandenburg, 2006 ▶); software used to prepare material for publication: *SHELXL97*.

## Supplementary Material

Crystal structure: contains datablock(s) global, I. DOI: 10.1107/S1600536812031066/nk2165sup1.cif

Structure factors: contains datablock(s) I. DOI: 10.1107/S1600536812031066/nk2165Isup2.hkl

Additional supplementary materials:  crystallographic information; 3D view; checkCIF report

## Figures and Tables

**Table 1:** Selected bond lengths (Å)

Cu—O1	1.935 (3)
Cu—N	1.984 (3)

**Table 2:** Hydrogen-bond geometry (Å, °)

*D*—H⋯*A*	*D*—H	H⋯*A*	*D*⋯*A*	*D*—H⋯*A*
C4—H4*A*⋯O2^ii^	0.93	2.58	3.460 (5)	159
C13—H13*A*⋯O3^iii^	0.93	2.58	3.284 (6)	133

Symmetry codes: (ii) 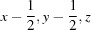; (iii) 
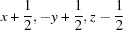
.

**Fig. 1.**
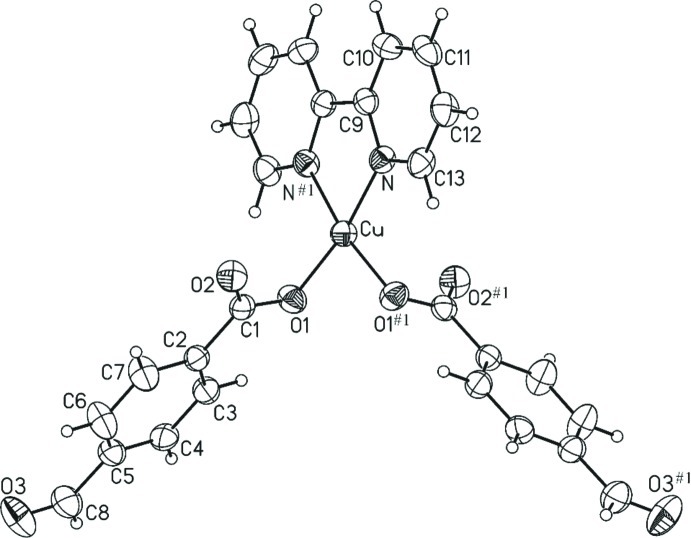
ORTEP view of the title compound, The dispalcement ellipsoids are drawn at 30% probability dispalcement ellipsoids. [Symmetry codes: (#1)-x + 1, y, -z + 1.5.]

**Fig. 2.**
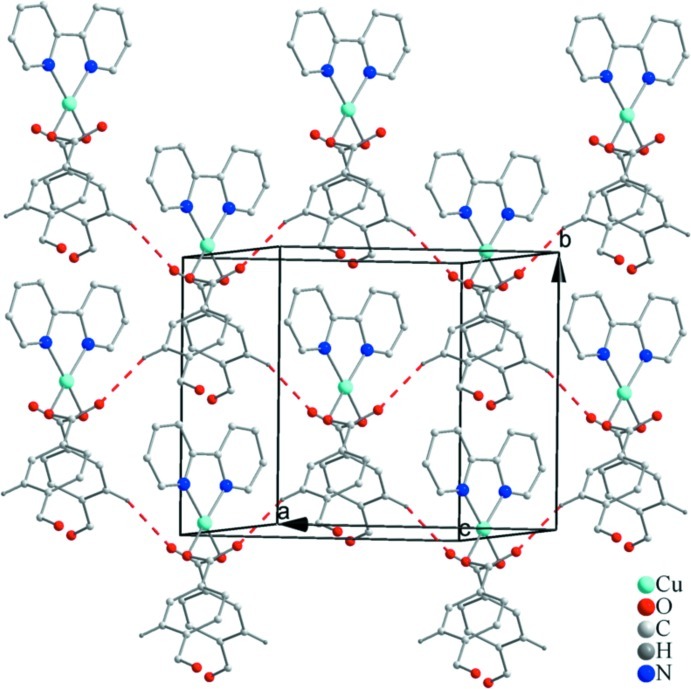
the two-dimensional lay structure parallel to (001).

## supplementary crystallographic information

### Comment

Transition metal complexes with carboxylic acids using various secondary building units (SBUs) frequently show interesting physical, chemical and/or biological properties (Sun *et al.*, 2002, Li *et al.*, 2007, Liu *et al.*, 2006). Herein, we are interested in selfassemblies of Cu^2+^ ions and 2,2'-bipydine (bipy) with 4-formylbenzoate, which led to the preparation of [Cu(C_10_H_8_N_2_)(C_8_H_5_O_3_)_2_].

The asymmetric unit contains a half Cu^II^ cation, a half bipy ligand and one 4-formylbenzoate (*L*^-^ = *p*-CHO-C_6_H_4_COO^-^) ligand. Both the Cu^II^ ion and bipy ligand lie on a crystallographic twofold rotation axis. In the complex, two crystallographically equivalent *L*^-^ anions function as monodentate ligands, while one bipy molecular functions as a terminal ligand adopting an expected chelating mode to coordinate with one Cu^II^ ion, forming a mononuclear unit. The Cu^II^ ion is coordinated by two nitrogen atoms (N and N^#1^, #1 = 1 - *x*, *y*, 1.5 - *z*) of one bipy ligand and two oxygen atoms (O1, O1^#1^) from two different carboxylic groups of two *L*^-^ ligands exhibiting essentially distorted square planar geometry (Fig.1). The Cu–N/O bonds in the quadrilateral plane are 1.984 (3) and 1.934 (3) Å, respectively. The *cisoid* bond angles fall in the region 80.9 (1)–93.8 (1) °, and *transoid* ones are both equal to 170.2 (1) °, exhibiting substantial deviations from 90 and 180 ° for a quadrate. In comparison with literatures, the above bonding values are normal (Li *et al.*, 2007).

The complex molecules are linked *via* weak C4–H4···O2^#2^ (#2 = -0.5 + *x*, -0.5 + *y*, *z*) hydrogen bonds to generate two-dimensional supramolecular layers in the *ab* plane. Along [001] direction the two-dimensional layers are stacked in a sequence ···ABABA··· and further connected *via* C13–H13···O3^#3^ (#3 = 0.5 + *x*, 0.5 - *y*, -0.5 + *z*) hydrogen bonds form three-dimensional supramolecular structure.

### Experimental

1 mL (1*M*) NaOH was added to an aqueous solution of CuCl_2_.2H_2_O (0.0852 g, 0.5 mmol) and Cl^-^ anions were removed by repeated centrifugation with NaOH, then 5.0 ml H_2_O and 5.0 ml EtOH were subsequently added. The blue suspension above was added to an aqueous ethanol solution (5.0 ml and 5.0 ml) of 4-formylbenzoic acid (0.1501 g, 1.0 mmol), then another aqueous ethanol solution (5.0 ml and 5.0 ml) of 2,2'-bipyridine (0.0782 g, 0.5 mmol) was added and stirred continuously for 1 h to give another blue suspension. After filtration, the blue filtrate (pH = 4.8) was allowed to evaporate at room temperature for one week to give dark blue plate-shaped crystals.

### Refinement

H atoms bonded to C atoms were placed in geometrically calculated position and were refined using a riding model, with *U*_iso_(H) = 1.2 *U*_eq_(C). After refinement there still remains one large residual peak 1.16 e.Å^-3^ high and located 2.36 Å from H10A. This was initially postulated as partly occupied water. However, the TG-DTA curve of the title complex shows no removal of a water molecula in the weight loss progress. We believe that the residual peak may be an artifact of poor crystal quality.

### Crystal data

**Table:**

[Cu(C_8_H_5_O_3_)_2_(C_10_H_8_N_2_)]	*F*(000) = 1060
*M**_r_* = 517.96	*D*_x_ = 1.459 Mg m^−^^3^
Monoclinic, *C*2/*c*	Mo *K*α radiation, λ = 0.71073 Å
Hall symbol: -C 2yc	Cell parameters from 11333 reflections
*a* = 11.923 (2) Å	θ = 3.2–27.4°
*b* = 10.992 (2) Å	µ = 0.97 mm^−^^1^
*c* = 18.275 (4) Å	*T* = 295 K
β = 100.11 (3)°	Block, blue
*V* = 2357.9 (8) Å^3^	0.23 × 0.17 × 0.08 mm
*Z* = 4	

### Data collection

**Table:**

Rigaku R-AXIS RAPID diffractometer	2700 independent reflections
Radiation source: fine-focus sealed tube	1672 reflections with *I* > 2σ(*I*)
Graphite monochromator	*R*_int_ = 0.067
ω scans	θ_max_ = 27.4°, θ_min_ = 3.2°
Absorption correction: empirical (using intensity measurements) (*ABSCOR*; Higashi, 1995)	*h* = −15→15
*T*_min_ = 0.820, *T*_max_ = 0.925	*k* = −14→14
11333 measured reflections	*l* = −23→23

### Refinement

**Table:**

Refinement on *F*^2^	Primary atom site location: structure-invariant direct methods
Least-squares matrix: full	Secondary atom site location: difference Fourier map
*R*[*F*^2^ > 2σ(*F*^2^)] = 0.058	Hydrogen site location: inferred from neighbouring sites
*wR*(*F*^2^) = 0.146	H-atom parameters constrained
*S* = 1.23	*w* = 1/[σ^2^(*F*_o_^2^) + (0.050*P*)^2^ + 1.7765*P*] where *P* = (*F*_o_^2^ + 2*F*_c_^2^)/3
2698 reflections	(Δ/σ)_max_ < 0.001
159 parameters	Δρ_max_ = 1.16 e Å^−^^3^
0 restraints	Δρ_min_ = −0.51 e Å^−^^3^

### Special details

**Table:**

Geometry. All e.s.d.'s (except the e.s.d. in the dihedral angle between two l.s. planes) are estimated using the full covariance matrix. The cell e.s.d.'s are taken into account individually in the estimation of e.s.d.'s in distances, angles and torsion angles; correlations between e.s.d.'s in cell parameters are only used when they are defined by crystal symmetry. An approximate (isotropic) treatment of cell e.s.d.'s is used for estimating e.s.d.'s involving l.s. planes.
Refinement. Refinement of *F*^2^ against ALL reflections. The weighted *R*-factor *wR* and goodness of fit *S* are based on *F*^2^, conventional *R*-factors *R* are based on *F*, with *F* set to zero for negative *F*^2^. The threshold expression of *F*^2^ > σ(*F*^2^) is used only for calculating *R*-factors(gt) *etc*. and is not relevant to the choice of reflections for refinement. *R*-factors based on *F*^2^ are statistically about twice as large as those based on *F*, and *R*- factors based on ALL data will be even larger.

### Fractional atomic coordinates and isotropic or equivalent isotropic displacement parameters (Å^2^)

**Table:**

	*x*	*y*	*z*	*U*_iso_*/*U*_eq_	
Cu	0.5000	0.53295 (6)	0.7500	0.0500 (3)	
O1	0.4216 (2)	0.4112 (2)	0.79856 (15)	0.0620 (7)	
O2	0.5705 (2)	0.4465 (3)	0.88645 (15)	0.0660 (8)	
O3	0.3550 (4)	−0.0045 (4)	1.1014 (2)	0.1132 (14)	
C1	0.4812 (3)	0.3924 (3)	0.8630 (2)	0.0505 (9)	
C2	0.4358 (3)	0.2966 (3)	0.9090 (2)	0.0496 (9)	
C3	0.3319 (3)	0.2404 (3)	0.8846 (2)	0.0571 (10)	
H3A	0.2888	0.2623	0.8391	0.069*	
C4	0.2918 (4)	0.1529 (3)	0.9271 (2)	0.0606 (11)	
H4A	0.2221	0.1158	0.9099	0.073*	
C5	0.3537 (4)	0.1197 (4)	0.9947 (2)	0.0597 (10)	
C6	0.4576 (4)	0.1749 (5)	1.0194 (3)	0.0834 (15)	
H6A	0.5006	0.1522	1.0648	0.100*	
C7	0.4980 (4)	0.2632 (4)	0.9774 (2)	0.0752 (13)	
H7A	0.5674	0.3007	0.9950	0.090*	
C8	0.3097 (5)	0.0272 (4)	1.0403 (3)	0.0819 (14)	
H8A	0.2411	−0.0099	1.0203	0.098*	
N	0.5875 (3)	0.6702 (3)	0.71745 (17)	0.0516 (8)	
C9	0.5513 (3)	0.7822 (3)	0.73221 (19)	0.0499 (9)	
C10	0.6077 (3)	0.8850 (4)	0.7152 (2)	0.0607 (11)	
H10A	0.5824	0.9618	0.7261	0.073*	
C11	0.7018 (4)	0.8726 (4)	0.6819 (2)	0.0677 (12)	
H11A	0.7411	0.9411	0.6704	0.081*	
C12	0.7375 (4)	0.7592 (5)	0.6655 (2)	0.0700 (12)	
H12A	0.8003	0.7493	0.6422	0.084*	
C13	0.6783 (3)	0.6600 (4)	0.6844 (2)	0.0619 (11)	
H13A	0.7025	0.5827	0.6737	0.074*	

### Atomic displacement parameters (Å^2^)

**Table:**

	*U*^11^	*U*^22^	*U*^33^	*U*^12^	*U*^13^	*U*^23^
Cu	0.0473 (4)	0.0495 (4)	0.0548 (4)	0.000	0.0132 (3)	0.000
O1	0.0622 (17)	0.0604 (16)	0.0625 (17)	−0.0078 (14)	0.0083 (15)	0.0070 (14)
O2	0.0510 (16)	0.077 (2)	0.0706 (18)	−0.0208 (15)	0.0118 (14)	−0.0027 (15)
O3	0.122 (3)	0.123 (3)	0.102 (3)	−0.006 (3)	0.040 (3)	0.049 (2)
C1	0.052 (2)	0.049 (2)	0.054 (2)	0.0002 (19)	0.0201 (19)	−0.0042 (18)
C2	0.050 (2)	0.050 (2)	0.051 (2)	−0.0035 (18)	0.0131 (18)	−0.0029 (17)
C3	0.059 (2)	0.058 (2)	0.053 (2)	−0.010 (2)	0.0038 (19)	−0.0009 (19)
C4	0.063 (3)	0.057 (2)	0.064 (3)	−0.020 (2)	0.015 (2)	−0.009 (2)
C5	0.066 (3)	0.054 (2)	0.063 (2)	−0.003 (2)	0.023 (2)	0.002 (2)
C6	0.076 (3)	0.107 (4)	0.065 (3)	−0.012 (3)	0.005 (2)	0.027 (3)
C7	0.060 (3)	0.095 (3)	0.066 (3)	−0.023 (3)	0.001 (2)	0.011 (3)
C8	0.093 (4)	0.078 (3)	0.083 (3)	−0.010 (3)	0.037 (3)	0.009 (3)
N	0.0466 (18)	0.0563 (18)	0.0545 (18)	0.0012 (15)	0.0155 (15)	−0.0035 (15)
C9	0.048 (2)	0.054 (2)	0.047 (2)	−0.0013 (18)	0.0053 (17)	0.0018 (17)
C10	0.060 (3)	0.053 (2)	0.068 (3)	−0.007 (2)	0.007 (2)	0.007 (2)
C11	0.063 (3)	0.073 (3)	0.066 (3)	−0.021 (2)	0.010 (2)	0.016 (2)
C12	0.056 (3)	0.092 (3)	0.066 (3)	−0.015 (3)	0.021 (2)	0.001 (3)
C13	0.056 (2)	0.070 (3)	0.063 (2)	−0.004 (2)	0.022 (2)	−0.007 (2)

### Geometric parameters (Å, º)

**Table:**

Cu—O1^i^	1.935 (3)	C6—C7	1.376 (6)
Cu—O1	1.935 (3)	C6—H6A	0.9300
Cu—N	1.984 (3)	C7—H7A	0.9300
Cu—N^i^	1.984 (3)	C8—H8A	0.9300
O1—C1	1.282 (4)	N—C13	1.334 (5)
O2—C1	1.229 (4)	N—C9	1.347 (4)
O3—C8	1.203 (6)	C9—C10	1.378 (5)
C1—C2	1.505 (5)	C9—C9^i^	1.482 (7)
C2—C3	1.386 (5)	C10—C11	1.374 (6)
C2—C7	1.386 (5)	C10—H10A	0.9300
C3—C4	1.374 (5)	C11—C12	1.367 (6)
C3—H3A	0.9300	C11—H11A	0.9300
C4—C5	1.373 (5)	C12—C13	1.375 (6)
C4—H4A	0.9300	C12—H12A	0.9300
C5—C6	1.382 (6)	C13—H13A	0.9300
C5—C8	1.469 (6)		
			
O1^i^—Cu—O1	92.48 (17)	C6—C7—C2	120.4 (4)
O1^i^—Cu—N	93.83 (12)	C6—C7—H7A	119.8
O1—Cu—N	170.15 (12)	C2—C7—H7A	119.8
O1^i^—Cu—N^i^	170.15 (12)	O3—C8—C5	125.5 (5)
O1—Cu—N^i^	93.83 (12)	O3—C8—H8A	117.3
N—Cu—N^i^	80.96 (18)	C5—C8—H8A	117.3
C1—O1—Cu	107.4 (2)	C13—N—C9	118.8 (3)
O2—C1—O1	123.3 (4)	C13—N—Cu	125.7 (3)
O2—C1—C2	121.3 (4)	C9—N—Cu	115.5 (3)
O1—C1—C2	115.5 (3)	N—C9—C10	121.2 (4)
C3—C2—C7	118.6 (4)	N—C9—C9^i^	113.9 (2)
C3—C2—C1	121.4 (3)	C10—C9—C9^i^	124.9 (2)
C7—C2—C1	120.0 (3)	C11—C10—C9	119.2 (4)
C4—C3—C2	120.7 (4)	C11—C10—H10A	120.4
C4—C3—H3A	119.6	C9—C10—H10A	120.4
C2—C3—H3A	119.6	C12—C11—C10	119.8 (4)
C5—C4—C3	120.6 (4)	C12—C11—H11A	120.1
C5—C4—H4A	119.7	C10—C11—H11A	120.1
C3—C4—H4A	119.7	C11—C12—C13	118.3 (4)
C4—C5—C6	119.1 (4)	C11—C12—H12A	120.8
C4—C5—C8	120.4 (4)	C13—C12—H12A	120.8
C6—C5—C8	120.5 (4)	N—C13—C12	122.7 (4)
C7—C6—C5	120.6 (4)	N—C13—H13A	118.6
C7—C6—H6A	119.7	C12—C13—H13A	118.6
C5—C6—H6A	119.7		

Symmetry code: (i) −*x*+1, *y*, −*z*+3/2.

### Hydrogen-bond geometry (Å, º)

**Table:**

*D*—H···*A*	*D*—H	H···*A*	*D*···*A*	*D*—H···*A*
C4—H4*A*···O2^ii^	0.93	2.58	3.460 (5)	159
C13—H13*A*···O3^iii^	0.93	2.58	3.284 (6)	133

Symmetry codes: (ii) *x*−1/2, *y*−1/2, *z*; (iii) *x*+1/2, −*y*+1/2, *z*−1/2.

## References

[bb1] Brandenburg, K. (2006). *DIAMOND* Crystal Impact GbR, Bonn, Germany.

[bb2] Higashi, T. (1995). *ABSCOR* Rigaku Corporation, Tokyo, Japan.

[bb3] Johnson, C. K. (1976). *ORTEPII* Report ORNL-5138. Oak Ridge National Laboratory, Tennessee, USA.

[bb4] Li, C. H., He, X. M., Yang, Y. Q. & Li, W. (2007). *Chin. J. Inorg. Chem.* **23**, 1449–1452.

[bb5] Liu, F. Q., Wang, Q. X., Jiao, K., Jian, F. F., Guang, Y. L. & Li, R. X. (2006). *Inorg. Chim. Acta*, **359**, 1524–1530.

[bb6] Rigaku (1998). *RAPID-AUTO* Rigaku Corporation, Tokyo, Japan.

[bb7] Rigaku/MSC (2004). *CrystalStructure* Rigaku/MSC Inc., The Woodlands, Texas, USA.

[bb8] Sheldrick, G. M. (2008). *Acta Cryst.* A**64**, 112–122. 10.1107/S0108767307043930 18156677

[bb9] Sun, J., Lin, J. L., Zheng, Y. Q. & Wang, J. Y. (2002). *J. Synth. Cryst.* pp. 365–369.

[bb10] Xu, W., Liu, W., Yao, F. Y. & Zheng, Y. Q. (2011). *Inorg. Chim. Acta*, **365**, 297–301.

